# Abnormally Elevated Follicle-Stimulating Hormone (FSH) Level in an Infertile Woman

**DOI:** 10.1155/2019/3071649

**Published:** 2019-09-22

**Authors:** Aurore Catteau, Kalyane Bach-Ngohou, Justine Blin, Paul Barrière, Thomas Fréour, Damien Masson

**Affiliations:** ^1^Laboratoire de biochimie, Institut de Biologie, CHU de Nantes, Nantes, France; ^2^Faculté de médecine, Université de Nantes, Nantes, France; ^3^Service de médecine et biologie du développement et de la reproduction, CHU de Nantes, Nantes, France

## Abstract

We report a case of 33-year-old woman with a 4-year primary infertility. A high isolated follicle-stimulating hormone (FSH) level was conflicting with the clinical situation and with other hormonal markers which were in favor of polycystic ovarian syndrome. We followed a strategy used to identify immune complexes involving FSH. The PEG precipitation test revealed that the high FSH level was almost exclusively due to the presence of autoimmune FSH immunoglobulin complex (macro-FSH). The profile obtained by gel filtration chromatography confirmed the presence of an FSH-immunoglobulin complex. Such immunological dysregulation could be explored in cases of unexplained infertility and recurrent IVF failure.

## 1. Introduction

The gonadotrophins, luteinizing hormone (LH) and follicle-stimulating hormone (FSH) are key regulators of the gonadal functions. Measurement of LH and FSH on day 3 of a spontaneous menstrual cycle, associated with E2 and AMH measurements, is essential to evaluate ovarian reserve in order to personalize ovarian stimulation protocol in women undergoing in vitro fertilization (IVF) [1]. Approximately 10%–20% of couples, which are unable to conceive, present an unexplained infertility with IVF failure [2]. In these cases, autoimmune mechanisms and production of autoantibodies can contribute to unexplained infertility. Elevated day 3 FSH levels can generally be observed in premature ovarian failure (POF) cases or gonadotrophin-secreting tumors. If the levels of FSH or LH are discordant with the clinical situation, the presence of anti-gonadotrophins should be evaluated by hormonal measurement after PEG precipitation and further confirmed by chromatography [3, 4]. This strategy and the procedures are equivalent to that used for the detection of a macroprolactin [4, 5].

## 2. Case Description

A 33-year-old woman was referred with her husband to the Department of Reproductive Medicine for a 4-year primary infertility. Both partners had no particular medical or surgical history, and physical examination was unremarkable. On the male side, sperm analysis was perfectly normal. On the female side, puberty started at 10 and menarche occurred at 16 years of age. Since then, this woman described major menstrual irregularity such as spaniomenorrhea or even amenorrhea. The antral follicle count (AFC) was high (approximately 40), strongly suggesting polycystic ovarian syndrome (PCOS) in accordance with Rotterdam criteria [6]. Overall hormonal assays performed are summarized in Table 1. Initial thyroid function tests revealed high thyroid-stimulating hormone (TSH) and normal free thyroxine level (T4L). Anti-thyroglobulin antibodies (AAT) were strongly positive. Serum estradiol (E2) and testosterone were found to be within normal ranges. Serum luteinizing hormone (LH) was moderately elevated. Serum anti-Müllerian hormone (AMH) level was remarkably high, in agreement with the PCOS diagnosis [7]. A remarkably high follicle-stimulating hormone (FSH) level was found (112 IU/L, normal range 1.5–13 IU/L), further confirmed in the next menstrual cycles with the same very elevated FSH value. Pituitary magnetic resonance imaging (MRI) was performed and was found normal. This very isolated FSH level was conflicting with the clinical situation and other hormonal markers which were in favor of PCOS.

For this patient, assay interference was suspected on the basis of discordance between the serum FSH and LH results and the clinical history. An interference in immunoassay is rare, but is very problematic because can drastically affect patient management. Endogenous immunoglobulins can be identified in this situation. Immunoglobulins can be directed against assay reagents or against analyte itself leading to the formation of macro-analyte corresponds to complex immunoglobulin-analyte. Interfering immunoglobulins can be identified by elimination with a polyethylene glycol precipitation and a specific identification. Like so, the analyte test is realized before and after treatment. If an interfering immunoglobulin is present, the recovery of analyte is reduced. A macro-analyte can be suspected if recoveries are normal (>40%) for others analytes [4].

In endocrinology, macro-hormone is frequently described for serum prolactin assays. If macro-prolactin is suspected, PEG precipitation is used to detect immunoglobulin interference [8]. Macroprolactin is biologically inactive but this presence is detected by all prolactin immunoassays and can lead to misdiagnosis. Collaboration between clinician and biologist can permit to suspect interference assays.

As this unexplained isolated high FSH level resembled macro-prolactin cases, we followed the same strategy in order to identify immune complexes involving FSH. Therefore, we measured FSH after polyethylene glycol (PEG) precipitation to screen for interference from autoimmune FSH immunoglobulin complex (macro-FSH). For this technique, 200 *µ*L PEG 6000 solution (25%) was mixed with 200 *µ*L sample, briefly vortex-mixed, and centrifuged for 15 min at 3000*g* at room temperature. FSH was then measured in the supernatant for recovery calculation. We used the same protocol to evaluate LH assays before and after PEG precipitation. The recovery rate of FSH was 12.1% and free-FSH (unprecipitated-FSH) level after PEG treatment was at the upper limit of the normal range (Table 1). LH assays have been also performed before and after PEG precipitation. Contrary to FSH, LH recovery following PEG precipitation was within normal ranges (>40%). No autoimmune LH immunoglobulin complex, macro-LH, was detected. This PEG precipitation test revealed that high FSH level in this patient was almost exclusively due to the presence of the high-molecular FSH form, the macro-FSH. The analysis of circulating FSH by gel filtration chromatography was subsequently performed using a column of Ultrogel® AcA 54. Serum was applied on the column and the fractions were collected for FSH determination.

**Figure 1:**
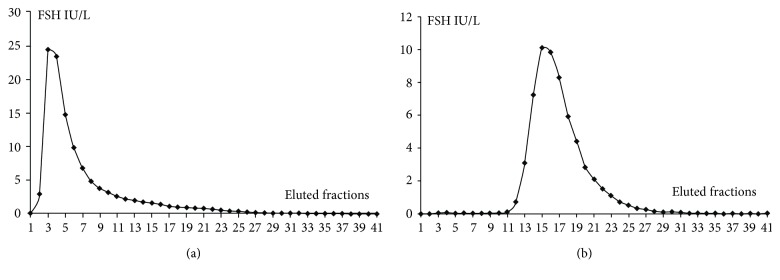
Gel filtration chromatography (GFC) elution profile. (a) Elution profile obtained with patient sample. (b) Elution profile obtained with normal control sample.

The gel filtration profile in this patient is represented in Figure 1(a). A high molecular form of FSH immunoreactivity was detected. This elution profile differed significantly from the pattern obtained in a normal control subject (Figure 1(b)). The high molecular mass peak corresponded to the molecular mass of an FSH-immunoglobulin complex. Additional analysis was performed. Serum was screened for ovarian autoantibodies by indirect immunofluorescence assay, but the result was negative. FSH is composed of two peptide subunits: alpha, common with LH, TSH, and hCG (human chorionic gonadotrophin), and beta that determines hormonal specificity. We wanted to evaluate if anti-FSH was directed against *α* or *β* subunit. An *α*-subunit assay and an hCG test by a *β*-subunit specific assay were realized (Table 1). The normal results indicated that these anti-FSH auto-antibodies were probably directed against *β*-subunit of FSH.

## 3. Discussion

We describe here a case of anti-FSH autoantibodies leading to persisting very high FSH serum levels in an infertile woman. FSH is a key regulator of the gonadal functions, along with LH. It is synthesized and released from the anterior pituitary gland as a heterodimeric glycoprotein of ~30 kDa molecular weight. It is composed of two peptide subunits: alpha, common with LH, TSH and hCG, and beta that determines hormonal specificity. Measurement of LH and FSH on day 3 of a spontaneous menstrual cycle, associated with E2 and AMH measurements, is essential to evaluate ovarian reserve in order to personalize ovarian stimulation protocol in women undergoing in vitro fertilization (IVF) [1]. Elevated day 3 FSH level can generally be observed in premature ovarian failure cases or gonadotrophin-secreting tumors [9, 10]. In this case, ovarian failure could be excluded because of elevated AFC and AMH levels. In our case, the description of menstrual irregularities such as spaniomenorrhea or even amenorrhea and an elevated AFC (approximately 40) are two Rotterdam criteria. The Rotterdam criteria define PCOS by the presence of at least two out of three criteria: oligo-anovulation, clinical and/or biochemical hyperandrogenism, and polycystic ovaries (≥12 follicles measuring 2–9 mm in diameter, or ovarian volume >10 ml in at least one ovary) [6]. A PCOS, a frequent endocrine disorder in women of reproductive age, is suspected for this patient. Moreover, AMH, a reliable marker of polycystic ovaries in PCOS, is here elevated [7, 11]. Furthermore pituitary MRI was normal, eliminating tumoral etiologies. Thus, premature ovarian failure and gonadotrophin-secreting tumor could be excluded. Therefore, another etiology was suspected for this isolated and persisting elevated FSH level.

Apart from PCOS, this patient presented a context of thyroid autoimmunity. This is frequent in women of reproductive age, and can be associated with female infertility and even negatively affect IVF outcome [12]. Furthermore, some studies have also suggested a connection between autoimmune thyroiditis and PCOS [13]. Several studies have described association between premature ovarian failure and autoimmune diseases such as adrenal autoimmunity, Hashimoto thyroiditis, diabetes mellitus, and rheumatoid arthritis. Among all autoimmune diseases associated with POF, thyroid disorders are the most common [14]. It has been proposed that reduced fertility might be associated with the dysregulation of immune system reactions resulting in enhancement of autoantibody production [15]. This autoimmunity increases miscarriage risks, reduces female fecundity and infertility treatment success. In case of an autoimmune disease, such as thyroid disorders, presence of anti-gonadotrophins (anti-LH, anti-FSH) is described in infertile women [14].

These antibodies have been identified in poor responders [14] and in cases of repeated implantation failure [15]. These observations suggested that these antibodies could inhibit either FSH or LH effects on folliculogenesis and ovulation *in vivo*, by interrupting the hormone-receptor binding or by increasing their clearance. An immunization against exogenous gonadotropins has been suggested to explain the presence of anti-FSH or anti-LH antibodies in women undergoing IVF, and may increase with repeated IVF cycles [16]. However, no exogenous FSH had been given to our patient before infertility work-up, ruling out this hypothesis. Consequently, anti-gonadotrophin autoimmunity may represent an interesting pathophysiological mechanism in POF.

Concerning anti-FSH, majority of these autoantibodies are directed against *β*-subunit of follicle-stimulating hormone [3]. These antibodies predominantly recognized a region of 16 amino-acids, probably representing the immunodominant epitope [17]. The *β*-subunit plays a role in FSH receptor binding. Therefore, the presence of anti-FSH directed against *β*-subunit can explain the infertility cases. In our patient, analyses were in favor of autoantibodies directed against *β*-subunit [18].

These autoantibodies deeply affect basal hormonal status and compel assisted reproductive technology (ART) specialists to adapt treatment strategies. Long term oestroprogestative contraceptive pill treatment has been suggested in order to suppress gonadotrophin secretion and consequently autoantibody production. Others advise an immune-modulating therapy such as glucocorticoid treatment for women with autoimmune diseases, anti-ovarian or anti-gonadotrophin antibodies in order to recover a satisfactory ovarian function and ultimately improve IVF outcome [15, 19]. However, these strategies have not been proven to be relevant yet in such cases, and the best protocol remains to be identified. In the case reported here, the couple was informed of these options and finally decided not to try these treatment. Concerning PCOS, eating right, exercising, and not smoking were very much suggested. In absence of hyperandrogenism, antiandrogen prescription was not necessary. Ovulation induction with clomiphene was proposed but the couple finally decided not to perform medically assisted procreation [20]. Treatment by L-thyroxine was started.

From an analytical point of view, anti-gonadotrophin antibodies can interfere with immunoassays via the formation of circulating hormone—immunoglobulin complexes [4]. The presence of heterophilic antibodies directed against antibodies of different animal species present in immunoassay [21], found in cases of rheumatoid arthritis for example, can also lead to falsely high or falsely low results in commonly used immunoassays such as FSH, LH, hCG or AMH. Important analytical steps should be followed when the presence of anti-gonadotrophins is suspected. Firstly, the same sample should be reanalyzed with the same assay, as the presence of antibody interference can give varying results when repeating the test. Secondly, hormonal measurement should be performed with an alternative assay. Indeed, the use of different antibodies in various immunoassays can lead to different sensitivity to analytical interference. Another strategy is to perform several dilutions. Serial dilutions of serum will differentiate true analyte from cross reactivity due to heterophile antibodies. Serial dilution of serum assays were made by dilution of the sample using the manufacturers' diluents. A nonlinear relationship is often obtained for serial dilutions in the presence of heterophile antibodies. However, it is possible to observe linear relationship in the serial dilutions in samples containing heterophile antibodies [22]. The presence of heterophilic antibodies can then be confirmed by adding irrelevant animal immunoglobulin to the sample prior to reassay, which may neutralize interfering antibodies. Finally, the presence of anti-gonadotrophins should be evaluated by hormonal measurement after PEG precipitation and further confirmed by chromatography. This strategy and the procedures are equivalent to those used for the detection of a macroprolactin [8].

Anti-FSH autoantibodies were identified in this patient referred for infertility workup and presenting with unexplained elevated isolated FSH levels. Such immunological dysregulation could be explored in cases of unexplained infertility and recurrent IVF failure. As anti-gonadotrophins can interfere with immunoassays, the dialog between biologist and clinician is obviously crucial to prevent from clinical misdiagnosis.

## 4. Conclusion

Isolated high basal FSH level can occur in a limited number of clinical situations, such as ovarian failure and gonadotrophin-secreting tumor. To identify the presence of anti-FSH autoantibodies, FSH measurement after PEG precipitation is recommended and should be confirmed by gel filtration chromatography. Anti-gonadotrophins were found in some infertile women and these antibodies could inhibit hormone activity in vivo and diminish efficacy of ovarian stimulation treatment. These autoantibodies must be screened in order to adapt treatment strategies and improve chances of pregnancy. The communication between the laboratory specialist and clinician is crucial to avoid clinical misdiagnosis.

## Figures and Tables

**Table 1:** Patients' results. (a) Serum hormonal concentrations on day 3 of a nonstimulated cycle. (b) Polyethylene Glycol precipitation (PEG) results.

Hormonal assays	Method	Patient	Normal reference range
(a) Day 3 of nonstimulated cycle			
LH (IU/L)	Elecsys® LH	18.6	2–11
FSH (IU/L)	Elecsys® FSH	112	1.5–13
E2 (pg/mL)	Cisbio estradiol	64.3	12–166
(pmol/L)	radioimmunoassay	236	44–609.2
hCG (IU/L)	Elecsys® hCG+*β*	<0.5	<5
AMH	Immunotech		
(ng/mL)	enzyme	9.4	2–6
(pmol/L)	immunoassay	67.1	14.3–42.8
TSH (mIU/L)	Elecsys® TSH	6.85	0.2–4
T4L (ng/L)	Elecsys® T4L	15.1	8.5–18
(pmol/L)		19.5	11–23.2
*α*-Subunit (IU/L)	Radioimmunoassay	0.19	0.02–0.90
AAT (U/ml)	Liaison® Anti-Tg	985	<100
Anti-ovarian Ab	Indirect immunofluorescence	Neg	Neg
Testosterone (ng/mL)	Cisbio testosterone radioimmunoassay	0.16	0.1–0.75
(nmol/L)		0.55	0.34–2.60

(b) After polyethylene glycol precipitation			
LH (IU/L)	Elecsys® LH	18.6	2–11
PEG unprecipitated LH (IU/L)		8.4	/
Recovery LH %		45.2	>40
FSH (IU/L)	Elecsys® FSH	112	1.5–13
PEG unprecipitated FSH (IU/L)		13.6	/
Recovery FSH %		12.1	>40

## Abbreviations

PCOS: Polycystic ovarian syndrome
TSH: Thyroid-stimulating hormone
T4L: Free thyroxine
AAT: Anti-thyroglobulin antibodies
E2: Estradiol
LH: Luteinizing hormone
AMH: Anti-Müllerian hormone
FSH: Follicle-stimulating hormone
MRI: Magnetic resonance imaging
PEG: Polyethylene glycol
macro-FSH: Autoimmune FSH immunoglobulin complex
hCG: Human chorionic gonadotrophin
IVF: Undergoing in vitro fertilization
POF: Premature ovarian failure.
